# Improved Cattle Disease Diagnosis Based on Fuzzy Logic Algorithms

**DOI:** 10.3390/s23042107

**Published:** 2023-02-13

**Authors:** Dilmurod Turimov Mustapoevich, Dilnoz Muhamediyeva Tulkunovna, Lola Safarova Ulmasovna, Holida Primova, Wooseong Kim

**Affiliations:** 1 Department of IT Convergence Engineering, Gachon University, Sujeong-Gu, Seongnam-Si 461-701, Gyeonggi-Do, Republic of Korea; 2 Tashkent Institute of Irrigation and Agricultural Mechanization Engineers, National Research University, Tashkent 100000, Uzbekistan; 3 Samarkand State University of Veterinary Medicine, Livestock and Biotechnologies, Samarkand 140103, Uzbekistan; 4 Samarkand Branch of Tashkent University of Information Technologies, Samarkand 140100, Uzbekistan; 5 Department of Computer Engineering, Gachon University, Sujeong-Gu, Seongnam-Si 461-701, Gyeonggi-Do, Republic of Korea

**Keywords:** osteodystrophy, secondary osteodystrophy, ketosis, hypomicroselementosis, decision making, expert systems, fuzzy sets

## Abstract

The health and productivity of animals, as well as farmers’ financial well-being, can be significantly impacted by cattle illnesses. Accurate and timely diagnosis is therefore essential for effective disease management and control. In this study, we consider the development of models and algorithms for diagnosing diseases in cattle based on Sugeno’s fuzzy inference. To achieve this goal, an analytical review of mathematical methods for diagnosing animal diseases and soft computing methods for solving classification problems was performed. Based on the clinical signs of diseases, an algorithm was proposed to build a knowledge base to diagnose diseases in cattle. This algorithm serves to increase the reliability of informative features. Based on the proposed algorithm, a program for diagnosing diseases in cattle was developed. Afterward, a computational experiment was performed. The results of the computational experiment are additional tools for decision-making on the diagnosis of a disease in cattle. Using the developed program, a Sugeno fuzzy logic model was built for diagnosing diseases in cattle. The analysis of the adequacy of the results obtained from the Sugeno fuzzy logic model was performed. The processes of solving several existing (model) classification and evaluation problems and comparing the results with several existing algorithms are considered. The results obtained enable it to be possible to promptly diagnose and perform certain therapeutic measures as well as reduce the time of data analysis and increase the efficiency of diagnosing cattle. The scientific novelty of this study is the creation of an algorithm for building a knowledge base and improving the algorithm for constructing the Sugeno fuzzy logic model for diagnosing diseases in cattle. The findings of this study can be widely used in veterinary medicine in solving the problems of diagnosing diseases in cattle and substantiating decision-making in intelligent systems.

## 1. Introduction

Cattle disease diagnosis is challenging due to similar symptoms between diseases. At present, the development of artificial intelligence (AI) via mathematical methods using fuzzy sets is essential in diagnosing and predicting animal diseases. Good research outcomes have been achieved in the development of computer diagnostic and prognostic systems to increase the quality of optimal treatment, early detection of types and causes of diseases, and targeted treatments. The improvement of these systems is required to enable the early diagnosis of animal diseases.

In cattle, osteodystrophy refers to a group of metabolic bone diseases that are characterized by abnormal bone growth and mineralization. There are numerous potential causes, such as mineral imbalances, hormonal disorders, and genetic predisposition. The symptoms in cattle may include lameness, stiffness, and decreased milk production.

Ketosis, also known as ketotic hypoglycemia, is a metabolic disorder that occurs when the cow’s body begins to break down fat instead of using carbs as its main source of energy. This can occur when the cow’s diet is too high in fat, when the cow is under stress, or when there is an increased demand for energy. The symptoms in cattle may include decreased appetite, weight loss, and less milk being produced.

Microelementosis, also known as trace mineral deficiency, is a condition caused by a lack of essential trace elements, such as copper, zinc, selenium and iodine, in the cow’s diet. It can lead to various health problems, such as immune dysfunction, reproductive failure, and poor growth. The symptoms in cattle may include anaemia, poor coat condition, and lower milk output.

Diseases with metabolic disorders are mainly diseases with disorders of carbohydrate, fat and protein metabolism, mineral metabolism disorders, and diseases with deficiency or excess of microelements. At the same time, there is a lack of minerals (macro and microelements) in the diet of animals, a violation of the glycogen-synthesizing function of the liver due to a sugar protein ratio below 0.8:1, ketonolactia, a decrease in the amount of hemoglobin, erythrocytes, total protein, nitrogen, and urea in the blood [1]. Eshburiev and Bell [1,2] have devoted their scientific activity to the study of diseases of ketosis, alimentary and secondary osteodystrophy, disorders of vitamin and mineral metabolism in animals and have come to different conclusions about the etiology, development mechanisms, diagnosis, treatments. and preventative measures for secondary osteodystrophy in dairy cows.

In addition, there are numerous scientific publications on the prevalence, causes, developmental features, clinical signs, diagnosis, treatment, and group prevention of primary and secondary osteodystrophy, ketosis, and microelementosis in cattle, while information on diseases in imported black-and-white cattle breeds and livestock bred in new farms is rare [3].

Mehmet [4] used a fuzzy logic (FL) method to determine whether an animal disease in the case of the possibility of an animal disease with neurological signs or reduced natural oddity is insufficiently calculated. Fuzzy sets can be applied to veterinary medicine to help determine the condition. Fuzzy sets allow for a more flexible and nuanced approach to decision-making as they account for the uncertainty and imprecision that often exists in medical diagnoses. By using fuzzy sets to evaluate the symptoms and test results of an animal, a veterinarian can perform a more informed and accurate diagnosis of the disease. This can lead to more effective treatment and improved outcomes for the animal.

Beata and Muhamedieva et al. [5,6] applied a k-nearest neighbor fuzzy classifier and pattern recognition theory to understand the abnormal breathing pattern resulting from diaphragm paralysis and identified the dominant component, tidal or frequency, of the breathing pattern on which lung ventilation is performed. This problem is considered in an experimental model of diaphragm paralysis due to bilateral phrenicotomy in drug-addicted, spontaneously breathing cats. From several recorded variables, two characteristics, minute ventilation and blood pressure, were selected and used for k-nearest neighbor analysis. The results showed that the ability to maintain ventilation significantly depends on the increase in the respiratory rate. Other breathing strategies proved ineffective. The assessment of the “k-nearest neighbor” based on two selected features caused it to be possible to determine the predominant breathing pattern with sufficient probability. Such an assessment can be a useful tool for predicting the development of compensatory strategies for respiratory disorders.

Rodrigo et al. [7] used three mathematical methods to determine the relationship between the weight and length of Cichla monoculus fish. The first method is an ordinary least squares regression, the second method uses raw data in nonlinear analysis, and the third is a combination of multivariate estimation and FL. The best option was sought among these methods. Although the nonlinear analysis provided better results than the least squares method, the best results were obtained with the help of fuzzy inference.

The significance of the correlation between fish length and weight and parameter interpretation was established by the Froese [8]. The condition factor, which was utilized to evaluate the relative health of each fish, was the parameter that was taken into consideration.

Barros et al. [9] investigated whether the knowledge component increased fish length when fish form or condition changed. This strategy does not address the issue of indicator variability. At least three variables, including environmental circumstances (such as the hydrological cycle season in big rivers with surrounding floodplains) and the stage of gonadal development at which the two populations are now, can be used to explain differences in average conditions for two populations. In reality, there was a general agreement that environmental factors, such the availability of food, significantly affect the condition factor. The change in the estimating technique does not take into account the intrinsic unpredictability of parameters, even though the approximation of nonlinear models should result in reliable parameter estimates.

In [10], the Takagi–Sugeno–Kanga neuro-fuzzy classifier was used to categorize three horse breeds (Jedju, Warmblood, and Thoroughbred) and four horse gaits (walk, sitting trot, rising trot, and gallop) from data that have been wavelet packetized. The fuzzy c-means clustering approach, which may address the issue of rising dimensionality due to flexible scatter partitioning, was used to construct the neuro-fuzzy classifier. In order to achieve this, the movement of a rider’s hip was employed as representative data for categorizing horse gait using data gathered by inertial sensors. Additionally, a training system that can be used in both real-world and virtual riding environments was created and a technique for examining rider movements was suggested. It was able to teach the right movement matching to a classed gait using the findings of rider analysis. The motion database was produced using information gathered from 17 inertial sensors connected to a motion capture suit worn by one of the top equestrian athletes in the nation. A variety of classifiers were tested in experiments using both raw and processed motion data to assess classification ability. The proposed approach outperformed a neural network classifier, a straightforward Bayesian classifier, and a network classifier using a radial basis function in terms of accuracy (97.5%) for the modified motion data.

Owing to the relevance of determining estrus for reproductive function, an algorithm was developed by Susana et al. [11] using fuzzy sets to predict estrus in dairy cows. Three input variables were used: (1) behaviors of dairy cows (raw, genital discharge, genital edema, frequent urination, and restlessness), (2) trying to sit on other cows, and (3) time since the last heat. The estrus detection rate, which is the percentage of correct estrus detection, was used as the output variable. The analysis was performed using various MATLAB 6.5 FL tools. The results showed that FL is a promising technique to predict estrus in dairy cows and can help in the decision-making process related to animal insemination.

Ferreira et al. [12] investigated how the effective detection of estrus in cows and heifers profoundly affects the reproductive performance of animals and profitability of livestock farms. Failure to detect estrus creates economic problems for farmers, mainly when artificial or controlled insemination is used. Even if a cow is in good breeding conditions, it is essential to correctly identify estrus to avoid the excessive use of hormones because herd productivity is considered adequate if dairy cows are farrowed once a year. Moreover, effective estrus detection is directly related to reproductive efficiency. To adequately determine estrus, it is necessary to evaluate the behavior of an animal, using the reproductive cycle of the animal as a starting point. Estrus is defined as the period when dry cows or heifers increase their reproductive hormone levels, which occurs every 18–24 days. The main characteristic of estrus is when a female agrees to the set, followed by other signals to help identify estrus, called secondary signals. Sexual behaviors comprise recognition (smell of the vulva, flehmen reflexes, accommodation-accompanying, and stalking), prematting (pressing the chin to the croup, headbutting, licking other parts of the body, and attempting to sit down), matting (attempt to mount, attempt to expose, attempt to mount with positive immobility, and maintenance or full mount), and a rest period.

Expert systems are computer programs that emulate the decision-making abilities of a human expert. They are designed to solve complex problems or make decisions in specific domains, such as medical diagnosis or financial planning. Pfeifer et al. [13] argue that expert systems and decision support systems (DSS) can complement each other in various ways.

One way is that a DSS can provide an expert system with a user-friendly interface, causing it to be more accessible to non-experts. Additionally, DSS can provide expert systems with additional data and analytical tools that can help the expert system make better decisions.

Another way is that expert systems can provide DSS with more accurate and reliable information, as they are able to process large amounts of data and make decisions based on specific knowledge and expertise. Pfeifer et al. [13] suggest that combining the strengths of expert systems and DSS can lead to more effective decision making.

To date, the issue remains of bridging the gap between the reasoning mechanism of a highly qualified doctor and the display of this process in the knowledge base and inference system in veterinary medicine. Further development requires the transition from formalisms that reflect the solution option that corresponds to the doctor’s logic in the educational version to the ability to extract and formalize difficult-to-verbalize representations, for example, intuitive or fuzzy for the expert in this field. The analysis of medical practice allows us to conclude that the incomplete and fuzzy presentation of the initial data characterizes the tasks of forecasting early differential diagnosis. There is also a high complexity of data formalization and the absence of a clear boundary for the values of diagnostic parameters. Considering the apparent fuzziness and inconsistency of medical information, it is evident that information technology is associated with great difficulties. One of the ways to formalize uncertainties and provide correct and adequate results is performing the methods of fuzzy mathematics. The use of the apparatus of fuzzy logic in solving these problems has great potential, which is confirmed in many studies reviewed.

A neutrosophic fuzzy set (NFS) is a mathematical construct that can be used to model the uncertainty and indeterminacy that often arises in real-world systems. It can provide a more accurate representation of reality by taking into account the fact that information is often incomplete or uncertain.

Recently, many works have proposed various algorithms and methods for single-valued neutrosophic fuzzy sets and interval neutrosophic fuzzy sets. In these works, some basic operations are introduced on single-valued and interval neutrosophic fuzzy sets, such as addition and multiplication, as well as some corresponding aggregation operators. Some basic operational laws of elementary neutrosophic fuzzy sets are defined, which include single-valued neutrosophic fuzzy sets and interval neutrosophic fuzzy sets [12].

Currently, FL is used in control and decision support systems, where the problem description approach cannot be accurate. A fuzzy inference system comprises output and input variables. For each variable, fuzzy sets that characterize it are expressed and a membership function is constructed for each fuzzy set. Afterward, rules that connect the output and input variables with the corresponding fuzzy sets are defined. The computational evaluation of a fuzzy inference system comprises fuzzification (building output variables that define a study), inferencing (applying fuzzy reasoning to fuzzy output data), and defuzzification (translating a linguistic value into a numerical value). Fuzzy reasoning can be obtained directly or indirectly.

In the listed works, we did not find the development of fuzzy logical algorithms for diagnosing diseases in cattle. In our research, we studied the prediction of the state of diseases of osteodystrophy, secondary acute dystrophy, ketosis, and hypomicroelementosis and built a model for predicting these diseases in cattle using fuzzy set theory and a new theory of neutrosophic fuzzy sets. 

In this article, based on 17 traits of cattle, membership functions will be built to diagnose the state of the diseases.

Based on laboratory data, the algorithm for constructing a fuzzy Sugeno model was improved for diagnosing diseases in cattle. In our proposed models, each input variable was calculated with its own membership functions with a fuzzy term (H, C, and B) used in the fuzzy inference equations of the Sugeno model. This will allow for determining the type of disease in cattle and performing an accurate diagnosis.

The paper is structured as follows. In Section 2, the materials and methods were analyzed. In Section 3, the informative features have been developed in the prediction of cattle diseases, whereas Section 4 introduced an Algorithm for constructing a fuzzy Sugeno model. The obtained results are described in Section 5. In Section 6, a discussion of the results is reported, as well as a comparison of the proposed solution with similar ones. Finally, conclusions are drawn in Section 7.

## 2. Materials and Methods

Our main tasks are to solve the problems of assessing the state of diagnostic models and highlighting which classification class the set of features belongs to. Such tasks are considered actual qualifying tasks of diagnostics.

The methods of fuzzy set theory and fuzzy logic are considered as an approach to the creation of an expert system that is capable of performing diagnostics taking into account data fuzziness. To date, there are a number of studies on medical systems that use fuzzy inferences. In most expert systems, confidence coefficients are used to represent the unreliability of information and are presented in the same way as in the system for diagnosing infectious diseases. This system contains about 450 rules that were developed with the help of the infectious disease group from Stanford University.

The complexity of a class structure, the vagueness of defined boundaries, and the significant overlapping areas cause doubt in the analysis of expert data in the field of veterinary medicine. The insufficiency of laboratory data significantly complicates the solution to the problem of complex systems for diagnosing cattle.

Scholars have shown that inference decision-making technologies of fuzzy sets have proven themselves well in diagnostic problems [14,15,16,17,18,19,20,21].

The difficulty in selecting the structural type and parameters of fuzzy decision rules is the main problem in the practical application of the task of the model of diagnosing cattle. The main part of this problem can be solved using a Sugeno fuzzy inference system [16,17,18]. Some fuzzy inference algorithms are the Mamdani algorithm, Sugeno algorithm, and ANFIS—adaptive fuzzy inference system (Adaptive-Network-Based Fuzzy Inference System).

When solving practical problems, the Mamdani algorithm is the most popular. Its popularity is due to its greater simplicity compared to other models.

A comparison of Sugeno and Mamdani fuzzy inference models shows that their difference lies in the knowledge base format and the defuzzification stage.

The main difference between such systems is that the Sugeno system produces a clear (quantitative) result in the form of a linear function value, while the Mamdani system produces a qualitative result (a fuzzy variable) [19,20]. If the Sugeno system is used as a mechanism for calculating a clear value, then the Mamdani algorithm can be used for linguistic analysis of the result [21].

The ANFIS network is a five-layer artificial neural network of direct signal propagation that uses the implementation algorithm.

The evaluation of a model using the theory of fuzzy sets is performed by polling an expert system and by developing a fuzzy rule “if… then….”. In this case, an expert is provided with sets of known values of input linguistic parameters suitable for diagnosis in some cases [22,23].

The following conditions must be met:

1. There is at least one rule for every linguistic term of the outgoing variable;

2. For any term unwanted variable, there is at least one rule that uses that term as a precondition (left side of the rule).

Otherwise, there will be an incomplete database of undefined rules. Suppose there are m rules in the database that look similar to this:

*R_1_: IF x_1_, is A_11_… AND… x_n_ is A_1n_, THEN Y is B_1_*

*…*

*R_i_: IF x_1_, is A_i1_… AND… x_n_ is A_in_, THEN Y is B_i_*

*…*

*R_m_: IF x_1_, is A_i1_… AND… x_n_ is A_mn_, THEN Y is B_m_,*

Here, *x_k_*, *k* = 1…*n* are input variables; *y*—output variables; and *A_ik_*—fuzzy sets with membership function. 

**Figure 1:**
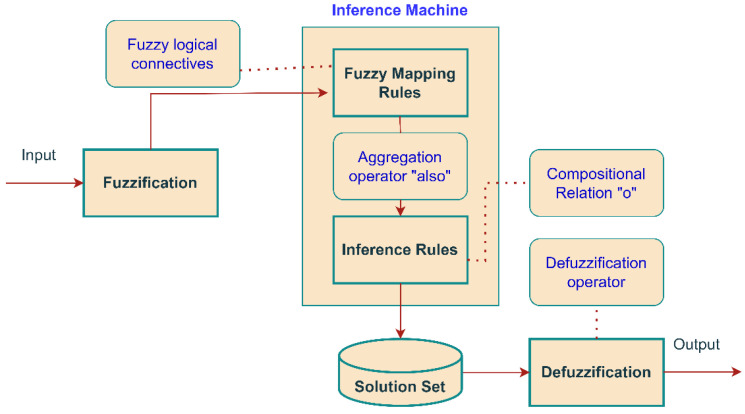
Fuzzy inference mechanism on the expert and control systems.

The construction of fuzzy logical inference is intended for the transition of input variables to output variables using a fuzzy knowledge base according to the following scheme, as shown in Figure 1 [23]:

The fuzzy inference mechanism used in various types of expert and control systems has a knowledge base formed by domain experts in the form of a set of fuzzy predicate rules of the following type:

1. Fuzziness (introduction of fuzziness, fuzzification). The membership functions defined for the input variables are applied to their true values to determine the truth level of each rule hypothesis.

2. Logical conclusion. The computed truth value for the assumptions of each rule is applied to the conclusion of each rule. This results in each summary variable for each rule are assigned to one fuzzy subset. Only min (minimum) or (multip) operations are usually used as inference rules. In the minimum logical conclusion, the inference relevance function is “cut off” at a height corresponding to the computed truth level of the rule’s assumptions (“AND” of fuzzy logic).

3. Composition. (In all rules), all fuzzy subsets assigned to each output variable are combined to form one fuzzy subset for each output variable. In such a combination, the max or sum operation is usually used. In the maximum composition, the combined output of a fuzzy subset is constructed as a point maximum over all fuzzy subsets (fuzzy logic “OR”).

4. In the conclusion (addition)—clarification (defuzzification) is used in cases where it is useful to change the form of a fuzzy set of conclusions to a clear figure. There are a large number of clarification methods, some of which are discussed below.

### The Developed Steps for Diagnosing Diseases in Cattle

**Figure 2:**
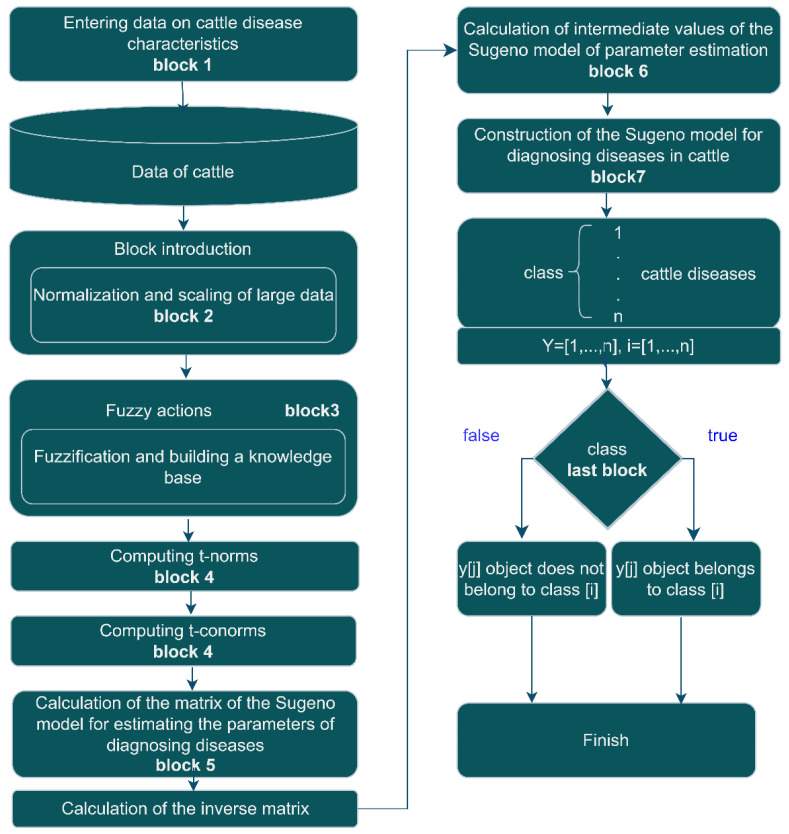
Functional scheme for diagnosing diseases in cattle.

The developed functional diagram for diagnosing diseases in cattle is presented in Figure 2.

In the first block, trait data for cattle diseases are entered, which are divided into classes.

In the second block, data with signs of cattle diseases are normalized and scaled.

On the third block, the fuzzy action of fuzzification and the construction of a knowledge base of fuzzy logic are carried out.

On the fourth block, the t-norm is calculated.

On the fifth block, the Sugeno matrix of the model for assessing the parameters of diagnosing diseases in cattle was calculated.

On the sixth block, the intermediate values of the Sugeno model for diagnosing diseases in cattle was calculated.

On the seventh block of building, the Sugeno model for diagnosing diseases in cattle was calculated.

On the last block, there is a classification cycle to which class the objects belong.

Based on the capabilities of veterinary diagnostic and prognostic tasks, it is appropriate to create decision rules as standard decision modules that are available in the nodes of the network system. The size of tasks solved by one mechanism is good if they are associated with the technological process of a single solution. For example, this applies for the processes of performing an inaccurate diagnosis according to the examination of a veterinarian; the process of determining the diagnosis with the evaluation of standard studies; the process of determining the diagnosis with the evaluation of data from special computer studies; etc.

## 3. Formation of Informative Features in the Prediction of Cattle Diseases

Ketosis, osteodystrophy, secondary osteodystrophy, and hypomicroelementosis in cattle were used as clinical indications (morphochemical and rumen contents) of non-communicable diseases in the creation of the membership function. The parameters used to evaluate the prevalence of non-contagious illnesses in cattle have been built into membership functions.

On the basis of these 17 traits of cattle, the duties of evaluating, categorizing, and predicting the sickness are resolved in this instance. The outcomes of a cow diagnosis can be categorized in the following ways, per established veterinary clinical practice:

**(a)**  **Clinical Status**

**One minute pulse in cattle.** We determine the membership function in the form of a triangle: the change in pulse per minute in cattle.

$$\mu \left(x_{2}\right)=\{\begin{array}{c} 0,\;if\;x\leq 50\\ \frac{x-50}{65-50},\;if\;50<x\leq 65\\ \frac{82-x}{80-65},\;if\;65<x\leq 80\\ 0,\;if\;x>80 \end{array}$$

The membership function is determined by the above formula; it is $\frac{x-50}{60-50}$ from 50 to 65 times per minute, $\frac{80-x}{80-65}$ from 65 to 80 times per minute, and 0 for up to 50 times per minute and more than 80 times per minute. An increase in the pulse per minute in cattle is the diagnosis of secondary osteodystrophy.

**Breath in one minute.** We determine the membership function using the breathing per minute in cattle.

$$\mu \left(x_{3}\right)=\{\begin{array}{c} 0,\;if\;x\leq 12\\ \frac{x-12}{18.5-12},\;if\;12<x\leq 18.5\\ \frac{25-x}{25-18.5},\;if\;18.5<x\leq 25\\ 0,\;if\;x>25 \end{array}$$

The membership function is determined by the above formula; it is $\frac{x-12}{18.5-12}$ from 12 to 18.5 times per minute, $\frac{25-x}{25-18.5}$ from 18.5 to 25 times per minute, and 0 for up to 12 times per minute and above 25 times per minute. A sign of an increase in breathing per minute is the diagnosis of ketosis and secondary osteodystrophy.

**Rumination in two minutes in cattle.** We determine the membership function using the change in rumination for two minutes in cattle.

$$\mu \left(x_{4}\right)=\{\begin{array}{c} 0,\;if\;x\leq 3\\ \frac{x-3}{4-3},\;if\;3<x\leq 4\\ \frac{5-x}{5-4},\;if\;4<x\leq 5\\ 0,\;if\;x>5 \end{array}$$

The membership function is determined by the above formula; it is $\frac{x-3}{4-3}$ from 3 to 4 times in two minutes, $\frac{5-x}{5-4}$ from 4 to 5 times in two minutes, and 0 for up to 3 times in two minutes and above 5 times in two minutes. An increase in rumination for two minutes in cattle is the diagnosis of secondary osteodystrophy and ketosis.

**(b)**  **Morphobiochemical factors**

**Erythrocyte.** We determined the membership function using the blood analysis of the erythrocyte index in cattle.

$$\mu \left(x_{5}\right)=\{\begin{array}{c} 0,\;if\;x\leq 5\\ \frac{x-5}{6.2-5},\;if\;5<x\leq 6.2\\ \frac{7.5-x}{7.5-6.2},\;if\;6.2<x\leq 7.5\\ 0,\;if\;x>7.5 \end{array}$$

The membership function is determined using the above formula; it is $\frac{x-5}{6.2-5}$ from 5 to 6.2 million/μL, $\frac{7.5-x}{7.5-6.2}$ from 6.2 to 7.5 million/µL, and 0 for up to 5 million/μL and above 7.5 million/μL. A decrease in the erythrocyte rate in the blood by 16%–33% in cattle is the diagnosis of osteodystrophy and hypomicroelementosis.

**Hemoglobin method (Sali’s Hemometer).** We determine the membership function using the hemoglobin method (Sali’s Hemometer) in cattle.

$$\mu (x_{6})=\{\begin{array}{c} 0,\;if\;x\leq 99\\ \frac{x-99}{114-99},\;if\;99<x\leq 114\\ \frac{114-x}{129-114},\;if\;114<x\leq 129\\ 0,\;if\;x>129 \end{array}$$

The membership function is determined by the above formula; it is $\frac{x-99}{114-99}$ from 99 to 114 g/L, $\frac{114-x}{129-114}$ from 114 to 129 g/L, and 0 for up to 99 g/L and above 129 g/L. A decrease in the hemoglobin concentration of cattle by 13–15% is the diagnosis of ketosis and secondary osteodystrophy.

**Total protein (using the refractometry method**). Total urine protein in cattle. We determine the membership function using the change in urine protein in cattle.

$$\mu \left(x_{7}\right)=\{\begin{array}{c} 0,\;if\;x\leq 68.3\\ \frac{x-68.3}{73.4-68.3},\;if\;68.3<x\leq 73.4\\ \frac{78.6-x}{78.6-73.4},\;if\;73.4<x\leq 78.6\\ 0,\;if\;x>78.6 \end{array}$$

The membership function is determined by the above formula; it is $\frac{x-68.3}{73.4-68.3}$ from 68.8 to 73.4 g/L, $\frac{78.6-x}{78.6-73.4}$ from 73.4 to 78.6 g/L, and 0 for up to 68.3 g/L and above 78.6 g/L. A decrease in the total protein in cattle by 21–75% is the diagnosis of secondary osteodystrophy, whereas an increase in the total protein by 86% is the diagnosis of ketosis.

**Total calcium (Vigev. Karakashov’s method).** We determine the membership function using the changes in calcium in cattle.

$$\mu \left(x_{8}\right)=\{\begin{array}{c} 0,\;if\;x\leq 2.5\\ \frac{x-2.5}{2.8-2.5},\;if\;2.5<x\leq 2.8\\ \frac{3.13-x}{3.13-2.8},\;if\;2.8<x\leq 3.13\\ 0,\;if\;x>3.13 \end{array}\;$$

The membership function is determined using the above formula; it is $\frac{x-2.5}{2.8-2.5}$ from 2.5 to 2.8 mmol/L, $\frac{3.13-x}{3.13-2.8}$ from 2.8 to 3.13 mmol/L, and 0 for up to 2.5 mmol/L and above 3.13 mmol/L. A decrease in the total calcium in the blood serum of cattle is the diagnosis of secondary osteodystrophy [24,25].

**The organic phosphorus (pulse method by V.F. Kromyslov and modification by L.A. Kudryatsev).** We determine the membership function using the organic phosphorus amount in cattle.

$$\mu \left(x_{9}\right)=\{\begin{array}{c} 0,\;if\;x\leq 1.45\\ \frac{x-1.45}{1.60-1.45},\;if\;1.45<x\leq 1.60\\ \frac{1.94-x}{1.94-1.60},\;if\;1.60<x\leq 1.94\\ 0,\;if\;x>1.94 \end{array}$$

The membership function is determined using the above formula; it is $\frac{x-1.45}{1.60-1.45}$ from 1.45 to 1.60 mmol/L, $\frac{1.94-x}{1.94-1.60}$ from 1.60 to 1.94 mmol/L, and 0 for up to 1.45 mmol/L and above 1.94 mmol/L. A small amount of organic phosphorus in the blood serum of cattle is used to diagnose osteodystrophy and secondary osteodystrophy.

**Glucose (color reaction with orthotoluidine).** We determine the membership function using the blood glucose amount in cattle.

$$\mu (x_{10})=\{\begin{array}{c} 0,\;if\;x\leq 2.2\\ \frac{x-2.2}{2.7-2.2},\;if\;2.2<x\leq 2.7\\ \frac{3.3-x}{3.3-2.7},\;if\;2.7<x\leq 3.3\\ 0,\;if\;x>3.3 \end{array}$$

The membership function is determined using the above formula; it is $\frac{x-2.2}{2.7-2.2}$ from 2.5 to 2.7 mmol/L, $\frac{3.3-x}{3.3-2.7}$ from 2.7 to 3.3 mmol/L, and 0 for up to 2.2 mmol/L and above 3.3 mmol/L. A low amount of glucose in the blood of cattle is low is used in the diagnosis of ketosis.

**Reserve alkali (I.P. Kondrakhin’s method).** We determine the membership function using the type of triangle: a change in the reserve alkali in cattle.

$$\mu \left(x_{11}\right)=\{\begin{array}{c} 0,\;if\;x\leq 44\\ \frac{x-44}{55-44},\;if\;44<x\leq 55\\ \frac{66-x}{66-55},\;if\;55<x\leq 66\\ 0,\;if\;x>66 \end{array}$$

The membership function is determined using the above formula; it is $\frac{x-44}{55-44}$ from 44 to 55, $\frac{66-x}{66-55}$ from 55 to 66, and 0 for up to 44 and above 66. A significantly low amount of alkaline reserve in the blood serum of cattle is used in the diagnosis of secondary osteodystrophy and ketosis.

**Copper.** We determine the membership function using the change in the amount of copper in the blood of cattle.

$$\mu \left(x_{12}\right)=\{\begin{array}{c} 0,\;if\;x\leq 14.1\\ \frac{x-14.1}{15.7-14.1},\;if\;14.1<x\leq 15.7\\ \frac{17.3-x}{17.3-15.7},\;if\;15.7<x\leq 17.3\\ 0,\;if\;x>17.3 \end{array}$$

The membership function is determined using the above formula; it is $\frac{x-14.1}{15.7-14.1}$ from 14.1 to 15.7 mmol/L, $\frac{17.3-x}{17.3-15.7}$ from 15.7 to 17.3 mmol/L, and 0 for up to 14.1 mmol/L and above 17.3 mmol/L. A decrease in the amount of copper in the blood of cattle is used in the diagnosis of osteodystrophy and hypomicroelementosis.

**Cobalt.** We determine the membership function using the change in cobalt in the blood of cattle.

$$\mu \left(x_{13}\right)=\{\begin{array}{c} 0,\;if\;x\leq 0.51\\ \frac{x-0.51}{0.68-0.51},\;if\;0.51<x\leq 0.68\\ \frac{0.85-x}{0.85-0.68},\;if\;0.68<x\leq 0.85\\ 0,\;if\;x>0.85 \end{array}$$

The membership function is determined using the above formula; it is $\frac{x-0.51}{0.68-0.85}$ from 0.51 to 0.68 mmol/L, $\frac{0.85-x}{0.85-0.68}$ from 0.68 to 0.85 mmol/L, and 0 for up to 0.51 mmol/L and above 0.85 mmol/L. A decrease in the amount of cobalt in the blood of cattle by 12% is used in the diagnosis of osteodystrophy and hypomicroelementosis.

**Manganese.** We determined the membership function using the change in the amount of manganese in the blood of cattle.

$$\mu \left(x_{14}\right)=\{\begin{array}{c} 0,\;if\;x\leq 2.73\\ \frac{x-2.73}{3.64-2.73},\;if\;2.73<x\leq 3.64\\ \frac{4.55-x}{4.55-3.64},\;if\;3.64<x\leq 4.55\\ 0,\;if\;x>4.55 \end{array}$$

The membership function is determined using the above formula; it is $\frac{x-2.73}{3.64-2.73}$ from 2.73 to 3.64 mmol/L, $\frac{4.55-x}{4.55-3.64}$ from 3.64 to 4.55 mmol/L, and 0 for up to 2.73 mmol/L and above 4.55 mmol/L. A decrease in the amount of manganese in the blood of cattle is used in the diagnosis of osteodystrophy and hypomicroelementosis.

**Zinc.** We determine the membership function using the change in zinc in the blood of cattle.

$$\mu \left(x_{15}\right)=\{\begin{array}{c} 0,\;if\;x\leq 46.2\\ \frac{x-46.2}{61.6-46.2},\;if\;46.2<x\leq 61.6\\ \frac{77.0-x}{77.0-61.6},\;if\;61.6<x\leq 77.0\\ 0,\;if\;x>77.0 \end{array}$$

The membership function is determined using the above formula; it is $\frac{x-46.2}{61.6-46.2}$ from 46.2 to 61.6 mmol/L, $\frac{77.0-x}{77.0-61.6}$ from 61.6 to 77.0 mmol/L, and 0 for up to 46.2 mmol/L and above 77.0 mmol/L. A decrease in the amount of zinc in the blood of cattle by 25–33% is used in the diagnosis of osteodystrophy and hypomicroelementosis.

**(c)**  **The scope of the scar**

**The number of ciliates in the rumen 1000/mL**. The number of ciliates in the rumen 1000/mL cattle.

$$\mu \left(x_{16}\right)=\{\begin{array}{c} 0,\;if\;x\leq 552\\ \frac{x-552}{595-552},\;if\;552<x\leq 595\\ \frac{638-x}{638-595},\;if\;595<x\leq 638\\ 0,\;if\;x>638 \end{array}$$

The membership function is determined using the above formula; it is $\frac{x-552}{595-552}$ from 552 to 595, $\frac{638-x}{638-595}$ from 595 to 638, and 0 for up to 552 and above 638. A decrease in the number of ciliates in the rumen by 300 ± 45 is used in the diagnosis of ketosis and hypomicroelementosis.

**Rumen fluid medium (Rameter).** We determine the membership function using the state of ruminal fluid in cattle.

$$\mu \left(x_{17}\right)=\{\begin{array}{c} 0,\;if\;x\leq 6.5\\ \frac{x-6.5}{7-6.5},\;if\;6.5<x\leq 7\\ \frac{7.5-x}{7.5-7},\;if\;7<x\leq 7.5\\ 0,\;if\;x>7.5 \end{array}$$

The membership function is determined using the above formula; it is $\frac{x-6.5}{7-6.5}$ from 6.5 to 7, $\frac{7.5-x}{7.5-7}$ from 7 to 7.5 mmol/L, and 0 for up to 6.5 and above 7.5. A decrease in the ruminal fluid status is used in the diagnosis of ketosis and secondary osteodystrophy.

## 4. Algorithm for Constructing a Fuzzy Sugeno Model

The etiology of secondary osteodystrophy, osteodystrophy, ketosis, and hypomicroelementosis using a prognostic model in veterinary practice causes it to be possible to optimize veterinary and sanitary work in the Samarkand region. The knowledge of existing experimental data causes it to be possible to increase the adequacy of a fuzzy expert system by considering the development of an algorithm for constructing the Sugeno fuzzy model.

This algorithm differs from other algorithms in that the confirming part of the rules does not use linguistic variables but linear expressions. Because of this, not all defuzzification methods are suitable for the algorithm. Sugeno’s [18] algorithm is more precise than Mamdani’s [24,25,26,27], which is sometimes difficult to implement.

The application of the Sugeno algorithm in constructing a fuzzy logical model for diagnosing diseases in cattle is used when linear expressions are known rather than the form of the output parameter correspondence function, each coefficient of the linear expression represents how each symptom of the disease affects when performing a diagnosis.

Unlike Mamdani’s algorithm, the rules containing disjunctions in the left parts of implications are not used. The advantages of the Sugeno algorithm lie in the lower labor intensity of calculations based on it, as well as in the ability to model very complex systems, the adequate description of which using the Mamdani scheme is almost impossible due to the extremely large number of emerging relationships between fuzzy parameters.

**Figure 3:**
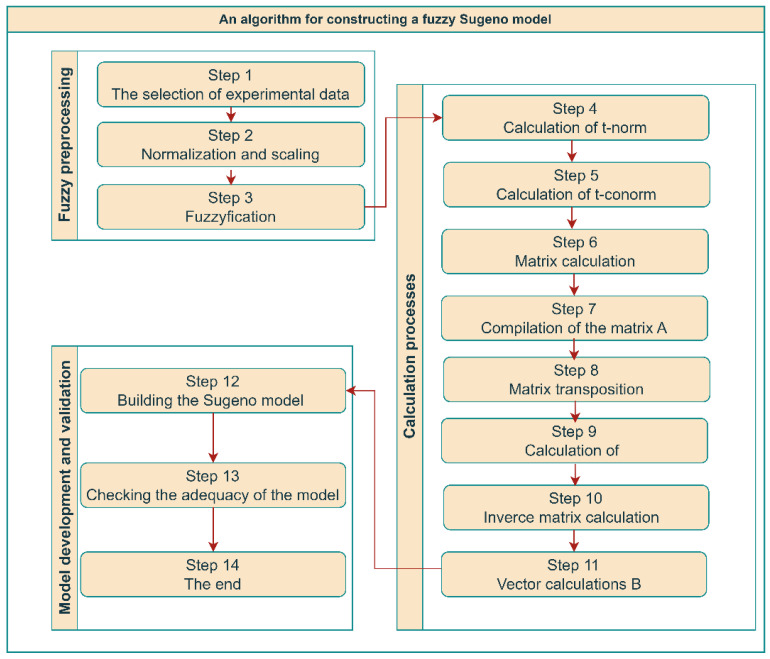
Fuzzy Sugeno model construction algorithm.

An algorithm for constructing a fuzzy Sugeno model is proposed (Figure 3).

Step 1. The selection of experimental data, $\left(X_{r},y_{r}\right),r=\bar{1,M}$, where $X_{r}=\left(\;x_{r,\;1},x_{r,\;2},\ldots ,x_{r,\;n}\right)$–input vector in r pair, and $y_{r}$ is the advisory output.

Step 2. Normalization and scaling $U_{i,j},$

(1) $$U_{i,j}=l\frac{x_{i,j}-\min(x_{i,j})}{\underset{j}{max}\left(x_{i,j}\right)-\underset{j}{min}\left(x_{i,j}\right)}$$

where l is the number of terms.

Step 3. Fuzzification,$\mu^{k}\left(U_{i,j}\right)=\frac{1}{1+{\left(\frac{U_{i,j}-C_{k}}{\sigma_{k}}\right)}^{2}}$, where $C_{k}=\bar{0,l}$, $k=\bar{1,l+1}$,$P_{i,\;j}=\left\{\;odds\;from\;max=\mu^{k}\;\left(U_{i,\;j}\right)\right\}$.

(2) $$\mu^{*}\;\left(U_{i,\;j}\right)=max\;\mu^{k}\left(\;U_{i,j}\right)=\frac{1}{1+{\left(\frac{x_{i,\;j}-P_{i,\;j\;}}{\sigma_{k}}\right)}^{2}},$$

where M is the number of rows and n is the number of columns, $i=\bar{1,M},j=\bar{1,n}$.

Step 4. Calculation of t-norm $mins=\left\{min\left(\mu \left(U_{i,j}\right)\right)\right\}$, where M is the number of rows and n is the number of columns, $i=\bar{1,M},j=\bar{1,n}$.

Step 5. Calculation of t-conorm, $\mu d_{j}\left(X_{r}\right)=\left\{max\left(mins\right)\right\}$, where M is the number of rows and m is the number of diseases, $r=\bar{1,m},j=\bar{1,M}$.

Step 6. Matrix calculation $\beta_{\;j,r},$

$$\beta_{j,r}=\frac{\mu d_{j}\left(X_{r}\right)}{\sum_{k=\bar{1,m}}\mu d_{k}\left(X_{r}\right)}$$

Step 7. Compilation of the matrix A,

$$A=\left[\begin{array}{c} \beta_{1,1},\ldots ,\beta_{1,m};\;X_{1,1}\;\beta_{1,1},\ldots ,X_{1,1}\;\beta_{1,m},\ldots ,X_{1,n}\;\beta_{1,1},\ldots ,X_{1,n}\;\beta_{1,m}\\ \beta_{M,1},\ldots ,\beta_{M,m};X_{M,1}\beta_{1,1},\ldots ,X_{M,1}\;\beta_{1,m},\ldots ,X_{M,n}\beta_{M,1},\ldots ,X_{M,n}\beta_{M,m} \end{array}\right]$$

Step 8. Matrix transposition A.

Step 9. Calculation of $A^{T}A$.

Step 10. Inverse matrix calculation ${\left(A^{T}A\right)}^{-1}$.

Step 11. Vector calculations B.

(3) $$B={\left(\;A^{T}\cdot A\right)}^{-1}\cdot \;A^{T}\cdot Y$$

Step 12. Building the Sugeno model.

Step 13. Checking the adequacy of the model.

(4) Y=A⋅B

Step 14. The end.

The membership functions of fuzzy terms used in this knowledge base were chosen by an expert.

Based on the proposed Sugeno fuzzy algorithm, a diagnostic program was created and a model for diagnosing diseases in cattle was developed [28,29,30].

## 5. Results

The application of signs or confidence levels can be shown using the following FL knowledge base for diagnosing diseases in cattle [31,32].

In the constructed fuzzy logical model for diagnosing diseases in cattle, a fuzzy classifier is used, which is a base of fuzzy rules.

The interpretability index characterizes the legibility and comprehensibility of terms, such as, for example, “if temperature is average, Pulse in one minute is high, Respiration in one minute is average, Rumination in two minutes is low, Red blood cell count is average, Hemoglobin is low, Total protein—medium, Total calcium—medium, Organic phosphorus—medium, Glucose—high, Reserve alkali—high, copper—medium, Cobalt—medium, Manganese—low, Zinc—low, The amount of ciliates in the rumen—medium, Condition of the scar fluid—low then cattle suffer from disease Etiology of secondary osteodystrophy”. The certifying part of the rules does not use linguistic variables but linear expressions, with each coefficient of the linear expression representing how each symptom of the disease affects the diagnosis.

To evaluate the linguistic values of the input parameters, the H-low level, B-high level, and C-medium level quantifiers were used.

The Sugeno FL model was built for diagnosing diseases in cattle from 17 main factors.

$x_{1}$: Temperature, °C;

$x_{2}$: Pulse, per minute;

$x_{3}$: Breath, per minute;

$x_{4}$: Rumination, per two minutes;

$x_{5}$: Number of erythrocytes mln/µL;

$x_{6}$: Hemoglobin, g/L (Sali’s Hemometer);

$x_{7}$: Total protein, g/L (by refractometry);

$x_{8}$: Total calcium, mmol/L (Vigev Karakashov’s method);

$x_{9}$: Organic phosphorous, mmol/L (pulse method by V.F. Kromyslov and modified by L.A. Kudryatsev);

$x_{10}$: Glucose, mmol/L (color reaction with orthotoluidine);

$x_{11}$: Reserve alkali ($CO_{2}$), % (I.P. Kondrakhin’s method);

$x_{12}$: Copper, mmol/L;

$x_{13}$: Cobalt, mmol/L;

$x_{14}$: Manganese, mmol/L;

$x_{15}$: Zinc, mmol/L;

$x_{16}$: The number of ciliates in the rumen 1000/mL;

$x_{17}$: Condition of cicatricial fluid (Rameter).

The FL model for diagnosing diseases in cattle is as follows [30].

Similar certainties are expressed in term sets, that is, in the form of linguistic truth values, among which one can single out the H-low level, B-high level, C-middle level, etc.

If the following conditions are true, then $y_{1}$ is calculated as follows:

IF ($x_{1}$ = C and $x_{2}$ = B and $x_{3}$ = C and $x_{4}$ = H and $x_{5}$ = C and $x_{6}$ = H and $x_{7}$ = C and $x_{8}$ = C $x_{9}$ = C and $x_{10}$ = B and $x_{11}$ = B and $x_{12}$ = C and $x_{13}$ = C and $x_{14}$ = H and $x_{15}$ = H and $x_{16}$ = C and $x_{17}$ = H);

Or IF ($x_{1}$ = H and $x_{2}$ = C and $x_{3}$ = C and $x_{4}$ = H and $x_{5}$ = C and $x_{6}$ = B and $x_{7}$ = C and $x_{8}$ = C and $x_{9}$ = B and $x_{10}$ = C and $x_{11}$ = C and $x_{12}$ = C and $x_{13}$ = C and $x_{14}$ = B and $x_{15}$ = C and $x_{16}$ = B and $x_{17}$ = C);

Or IF ($x_{1}$ = B and $x_{2}$ = C and $x_{3}$ = C and $x_{4}$ = H and $x_{5}$ = B and $x_{6}$ = C and $x_{7}$ = B and $x_{8}$ = B and $x_{9}$ = H and $x_{10}$ = C and $x_{11}$ = C and $x_{12}$ = B and $x_{13}$ = B and $x_{14}$ = H and $x_{15}$ = B and $x_{16}$ = B and $x_{17}$ = C);

Or IF ($x_{1}$ = B and $x_{2}$ = C and $x_{3}$ = B and $x_{4}$ = C and $x_{5}$ = B and $x_{6}$ = H and $x_{7}$ = H and $x_{8}$ = B and $x_{9}$ = C and $x_{10}$ = C and $x_{11}$ = H and $x_{12}$ = B and $x_{13}$ = B and $x_{14}$ = H and $x_{15}$ = C and $x_{16}$ = H and $x_{17}$ = C);

Or IF ($x_{1}$ = C and $x_{2}$ = C and $x_{3}$ = C and $x_{4}$ = C and $x_{5}$ = B and $x_{6}$ = C and $x_{7}$ = B and $x_{8}$ = H and $x_{9}$ = B and $x_{10}$ = C and $x_{11}$ = H and $x_{12}$ = B and $x_{13}$ = C and $x_{14}$ = C and $x_{15}$ = B and $x_{16}$ = C and $x_{17}$ = B)

Then

(5) $$\begin{array}{l} y_{1}=2.86-0.030x_{1}-0.009x_{2}-0.028x_{3}-0.019x_{4}+0.049x_{5}+0.0095x_{6}-0.007x_{7}+0.052x_{8}+\\ \;1.49x_{9}+0.46x_{10}+0.049x_{11}+0.126x_{12}-0.94x_{13}-1.60x_{14}-0.045x_{15}+0.009x_{16}-0.317x_{17} \end{array}$$

Here, $y_{1}$ is output, which means the disease osteodystrophy.

$y_{2}$ is determined as follows if the following statements are true:

IF ($x_{1}$ = H and $x_{2}$ = B and $x_{3}$ = C and $x_{4}$ = H and $x_{5}$ = C and $x_{6}$ = H and $x_{7}$ = C and $x_{8}=\;Bx_{9}$ = H and $x_{10}$ = C and $x_{11}$ = H and $x_{12}$ = C and $x_{13}$ = C and $x_{14}$ = H and $x_{15}$ = C and $x_{16}$ = C and $x_{17}$ = B);

Or IF ($x_{1}$ = B and $x_{2}$ = C and $x_{3}$ = C and $x_{4}$ = B and $x_{5}$ = B and $x_{6}$ = B and $x_{7}$ = C and $x_{8}$ = C and $x_{9}$ = C and $x_{10}$ = C and $x_{11}$ = H and $x_{12}$ = B and $x_{13}$ = B and $x_{14}$ = H and $x_{15}$ = C and $x_{16}$ = B and $x_{17}$ = C);

Or IF ($x_{1}$ = B and $x_{2}$ = C and $x_{3}$ = H and $x_{4}$ = C and $x_{5}$ = C and $x_{6}$ = H and $x_{7}$ = B and $x_{8}$ = C and $x_{9}$ = C and $x_{10}$ = C and $x_{11}$ = C and $x_{12}$ = B and $x_{13}$ = C and $x_{14}$ = B and $x_{15}$ = B and $x_{16}$ = C and $x_{17}$ = H);

Or IF ($x_{1}$ = C and $x_{2}$ = H and $x_{3}$ = B and $x_{4}$ = C and $x_{5}$ = C and $x_{6}$ = H and $x_{7}$ = B and $x_{8}$ = C and $x_{9}$ = C and $x_{10}$ = C and $x_{11}$ = C and $x_{12}$ = B and $x_{13}$ = C and $x_{14}$ = B and $x_{15}$ = B and $x_{16}$ = C and $x_{17}$ = H);

Or IF ($x_{1}$ = B and $x_{2}$ = C and $x_{3}$ = C and $x_{4}$ = C and $x_{5}$ = H and $x_{6}$ = C and $x_{7}$ = H and $x_{8}$ = C and $x_{9}$ = B and $x_{10}$ = C and $x_{11}$ = B and $x_{12}$ = C and $x_{13}$ = B and $x_{14}$ = B and $x_{15}$ = B and $x_{16}$ = C and $x_{17}$ = B)

Then

(6) $$\begin{array}{l} y_{2}=5.37+0.02x_{1}+0.006x_{2}+0.02x_{3}+0.01x_{4}-0.10x_{5}-0.002x_{6}+0.007x_{7}-0.02x_{8}-1.17x_{9}\\ \;-0.35x_{10}-0.03x_{11}-0.20x_{12}+0.03x_{13}+0.99x_{14}+0.015x_{15}-0.01x_{16}+0.21x_{17}. \end{array}$$

Here, $y_{2}$ means secondary osteodystrophy.

The presented methodology for constructing a Sugeno FL model and the use of neutrosophic fuzzy sets in solving problems of diagnosing diseases in cattle is one of the modern specializations in the field of AI and is aimed at creating methods for solving problems in many fields of knowledge, bringing computational solutions closer to human solutions.

An algorithm for fuzzy inference is developed and the dependence of parameters is considered. To fully introduce fuzzy information, we developed a fuzzy algorithm that uses fuzzy arithmetic in fuzzy inference, which will yield the least loss of information comprising uncertainties in a computational experiment [33,34,35,36].

The results of a computational experiment using our developed diagnostic program based on a fuzzy rule for classifying, evaluating, and predicting the states of weakly formalized processes are presented.

The problems of classification and evaluation are solved with the help of the developed program; a comparative analysis is performed between the results of the proposed algorithm and some existing algorithms.

## 6. Discussion

As a result, the classification errors from 2% to 14% were observed during the classification process. When this issue was resolved based on the Sugeno FL model, the level of error in it became much larger. Table 1 shows the results obtained at different levels based on the proposed Sugeno model and the method of group accounting for arguments and the mathematical model of E. Shortleaf. The results show the high efficiency of decision-making algorithms for predicting, classifying, and measuring weakly formalized processes described using fuzzy models [28,29].

For comparative analysis, well-known model problems are used, located at the electronic address: http://www.ics.uci.edu/~mlearn/databases/. These include the following tasks: Ecoli Data Set, Hepatitis C Virus (HCV) for Egyptian patients, and Breast Cancer Coimbra task. Table 2 shows the parameters of the listed tasks and the proposed task below. Table 3 shows comparative analysis of the proposed model with other databases.

**Figure 4:**
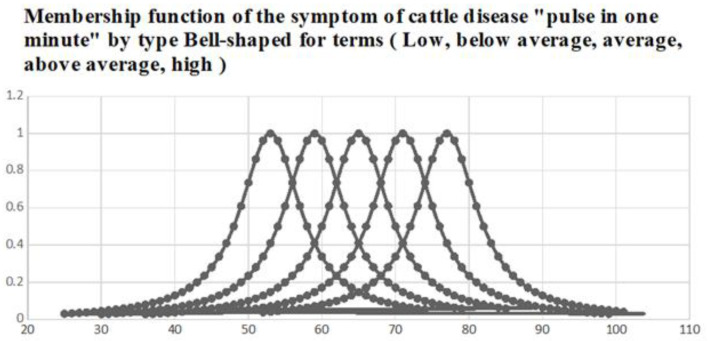
Membership function of output terms before learning.

The membership functions of fuzzy terms used in the fuzzy knowledge base chosen by experts are shown in Figure 4.

The membership functions of fuzzy terms after training are shown in Figure 4.

**Figure 5:**
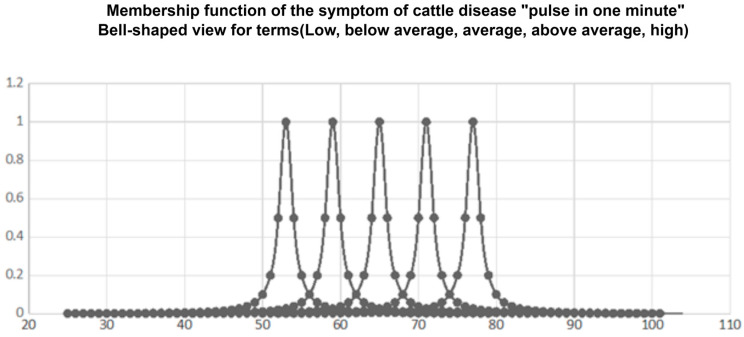
Membership functions of input terms after learning.

From Figure 5, as a result of training the neuro-fuzzy network, the parameters with the membership function have undergone the greatest changes:

(7) $$\mu \left(x_{i}\right)=\frac{1}{1+{\left(\frac{x_{i}-b}{\sigma_{i}}\right)}^{2}},$$

whereas other parameters remained virtually unchanged. This is explained by the fact that, when forming the fuzzy knowledge base, the expert quite accurately indicated the positions of the maxima of the membership functions (parameters b). The expert’s choice of large values of parameter c indicates significant uncertainty in the evaluation of fuzzy terms. Decreasing the values of parameter c while learning caused a “concentration” (compression) of membership functions, which indicates the removal of uncertainty in the estimates of fuzzy terms.

Thus, a method for representing linguistic knowledge about the object of identification is considered.

The obtained results show that our program can be used in forecasting, diagnostics, situational management, multivariate analysis, automatic classification, and other tasks of expert information processing.

## 7. Conclusions

The following conclusions were drawn from the conducted research:

1. The membership functions of the parameters for diagnosing the state of disease in cattle were built based on 17 signs.
2. The algorithm for creating a fuzzy Sugeno model for diagnosing diseases in cattle, based on laboratory data, is developed. Each input variable in our suggested models was computed using its own membership functions together with a fuzzy term (H, C, or B) employed in the Sugeno model’s fuzzy inference equations. This will enable identifying the disease’s type in cattle and providing a precise diagnosis.
3. As a result of our analysis, a fuzzy inference decision rule for diagnosing diseases in cattle was developed. This caused it to be possible to develop algorithms for constructing an FL model using the results of the experimental tests performed on cattle.
4. Based on the expert system, a Sugeno fuzzy logical model was built for diagnosing diseases in cattle. The analysis of the adequacy of the results obtained using the fuzzy Sugeno model was carried out. The analysis showed that the result obtained using the Sugeno model provides a diagnosis error of 2%.

Some limitations were noted during the study. When solving the problems of building a model for diagnosing diseases in cattle, the number of adjustable parameters is less than the volume of the data sample m∗(n+1)<M, so the equation Y=A∗B  does not have an exact solution. In this case, the solution can be found using the pseudo-inversion of the matrix $A:B={\left(A^{T}\cdot A\right)}^{-1}\cdot A^{T}\cdot Y.\;$ However, the problems of finding a solution to this are related to the possible singularity of the matrix ${\left(A^{T}\cdot A\right)}^{-1}$. In future studies, we will continue to solve this problem.

Future tasks include increasing the accuracy of the method and developing a small real-time model with reliable cattle disease detection performance using AI-based [37,38,39,40,41,42] and computer vision approaches [43,44,45,46,47,48,49].

## Figures and Tables

**Table 1:** Sugeno FL model and comparative results with the method of group accounting of arguments and the mathematical model of E. Shortleaf.

Model	Number of Objects	Number of Classes	Result
Sugeno FL model	100	4	98.8%
Method of group accounting of arguments	100	4	92.8%
Mathematical model of E. Shortlif	100	4	86.4%

**Table 2:** Parameters of model tasks and the proposed model.

Title Data Set	Number of Class	Number of Factors	Number of Objects
Escherichia coli	7	8	336
Hepatitis C Virus (HCV) for Egyptian patients	4	19	1385
Breast Cancer Coimbra	2	10	116
Cattle	4	17	100

**Table 3:** Comparative analysis of the proposed model with other databases.

	Sugeno Algorithm			Method of Group Accounting of Arguments		
	Poor	Medium	High	Poor	Medium	High
Hepatitis C Virus (HCV) for Egyptian patients	95.4	96.5	99.1	82.4	83.7	86.1
Breast Cancer Coimbra	95.2	96.1	98.8	84.4	86.4	89.1
E.coli (Escherichia coli)	93.5	98.8	98.8	86.4	92.3	92.8

## Data Availability

Data sharing not applicable.
